# *Streptomyces flavusporus* sp. nov., a Novel Actinomycete Isolated from Naidong, Xizang (Tibet), China

**DOI:** 10.3390/microorganisms13051001

**Published:** 2025-04-27

**Authors:** Dan Tang, Xiaoxia Zhou, Haolin Qian, Yu Jiao, Yonggang Wang

**Affiliations:** School of Life Science and Engineering, Lanzhou University of Technology, Lanzhou 730050, China; 18238719396@163.com (X.Z.); 18264821573@163.com (H.Q.); 15569965819@163.com (Y.J.); 412316788@163.com (Y.W.)

**Keywords:** *Streptomyces*, biosynthetic gene cluster, taxonomy and nomenclature, actinomycetes, extreme environments

## Abstract

The exploration of *Streptomyces* from extreme environments presents a particularly compelling avenue for novel compound discovery. A Gram-positive, pink-pigmented *Streptomyces* strain designated HC307^T^ was isolated from a soil sample collected in Xizang (Tibet), China. The exploration of *Streptomyces* from extreme environments presents a particularly compelling avenue for novel compound discovery. In this study, the 16S rRNA sequence of strain HC307^T^ exhibited the highest similarity with *Streptomyces prasinosporus* NRRL B-12431^T^ (97.5%) and *Streptomyces chromofuscus* DSM 40273^T^ (97.3%), which were below 98.7%. The draft genome of the bacteria was 10.0 Mb, with a G+C content of 70.0 mol%. The average nucleotide identity (ANI) values of strain HC307^T^ and similar type strains ranged from 78.3% to 87.5% (<95%). The digital DNA-DNA hybridization (dDDH) values ranged from 22.6% to 33.9% (<70%), which was consistent with the results obtained from phylogenetic tree analysis. Phenotypically, this bacterium grew within the temperature range of 25–40 °C, at a pH range of 5 to 9, and in NaCl concentrations from 0% to 6% (*w*/*v*). The polar lipid profile of strain HC307^T^ was diphosphatidylglycerol, phosphatidylglycerol, phosphatidylethanolamine and unidentified lipids. The analysis of 32 biosynthetic gene clusters (BGCs) indicated the strain’s capacity to synthesize diverse compounds. Phylogenetic and phenotypic analyses demonstrated that strain HC307^T^ represented a novel species within the genus *Streptomyces*, and proposed the name *Streptomyces flavusporus* sp. nov., with strain HC307^T^ (=DSM 35222^T^=CGMCC 32047^T^). The strain was deposited in Deutsche Sammlung von Mikroorganismen und Zellkulturen and the China General Microbiological Culture Collection Center for patent procedures under the Budapest Treaty.

## 1. Introduction

The genus *Streptomyces* is the largest and most important within the family *Streptomycetaceae*, serving as its type genus. First described by Waksman and Henrici in 1943, the genus *Streptomyces* stands alone as the sole member of the family *Streptomycetaceae* [1,2]. *Streptomyces*, a genus of actinomycetes, is a Gram-positive bacterium distinguished by its complex hyphal system and a high GC content in its genome [3,4]. The distribution of *Streptomyces* is shaped by a range of biological and physicochemical factors, leading to their presence in marine environments [5], soils, rhizospheres, animal excreta and some Endophytic *Streptomyces* [3].The List of Prokaryotic names with Standing in Nomenclature (LPSN) currently lists 743 *Streptomyces* (https://lpsn.dsmz.de/genus/streptomyces) which are child taxa with a validly published and correct name, accessed on 1 March 2025 [6].

As the primary source of antibiotics, nearly 80% of the naturally occurring antibiotics are produced by *Streptomycetes* [7,8]. Staurosporine, a novel compound isolated from *Streptomyces* sp. LY1209, demonstrated antiproliferative effects on Molt 4 cells. Furthermore, it was identified as a putative tyrosine kinase inhibitor, indicating its potential for therapeutic intervention in acute lymphoblastic leukemia [9]. Salinamides A-F, a class of peptide antibiotics isolated from *Streptomyces* sp. CNB091, demonstrated significant antimicrobial and ribonuclease inhibitory activity [10]. However, the overuse and depletion of antibiotics have led to chronic infections and antibiotic resistance [11]. Consequently, the screening of new antibiotics from *Streptomyces* has become an interesting challenge. However, the probability of discovering novel antibiotics from traditional methods used to select actinomycetes was less than one in a million [12], and known compounds were frequently re-isolated [13,14]. In 2011, cyclo-(tryptophanyl-prolyl) was initially isolated from a *Streptomyces* sp. and exhibited broad-spectrum antibacterial activity [15], while in 2016 it was isolated again from *Streptomyces* sp. SUK 25 with good antimicrobial potential against MRSA [16].

The exploration of *Streptomyces* in unique physiological environments holds promise for the development of novel antibiotics or pharmaceuticals [17,18]. The primary reason is that extreme environments can stimulate the expression of silent gene clusters [19]. For instance, high-throughput screening of lichen samples from Qinghai Lake had revealed that *Streptomyces* isolated from these samples exhibited broad-spectrum antimicrobial activities [20]. Notably, the identified compounds include anthracyclines, which possess significant antitumor and antibacterial properties and were commonly used in combination chemotherapy for cancer [5]. The Naidong region of Xizang (Tibet) is a semi-arid area with a harsh environment featuring high altitudes, strong solar radiation, low temperatures, and low oxygen levels due to thin air. It also undergoes severe drought-flood changes and extreme weather occurrences. Therefore, the identification of novel species from unique habitats provides a foundation for the discovery of secondary metabolites.

In this study, we identified 33 isolates of *Streptomycetes* from the barren soil of the Naidong area in Xizang (Tibet), one of which, strain HC307^T^, had low 16S rRNA similarity. The taxonomic position of the strain was determined through a combination of phenotypic, 16S rRNA gene and genomic characterization. In addition, the profiling of BGCs demonstrated that the strain exhibited the potential for the biosynthesis of antimicrobial compounds. The application of genomics and synthetic biology techniques can promote the expression of related gene clusters and lay a foundation for the identification of new chemicals.

## 2. Materials and Methods

### 2.1. Isolation and Cultivation Conditions

The sample was collected from the wasteland soil in Naidong District, Shannan Prefecture, Xizang (Tibet) (N 29°29′38″, E 91°68′91″), China, at an altitude of 3650 m. During the study, dead leaves and weeds were removed from the ground surface, and the top layer (0–20 cm) of soil was collected as test sample [21]. For each sample plot, one sampling point was taken as the center and radiated 5 m around it, and the radial periphery was sampled. Totally 9 samples were collected, and every 3 copies were mixed, which were considered as 3 biological replicates. The collected samples were stored in sterile self-sealing bags. The samples were quickly brought back to the laboratory for preservation at −80 °C for bacterial diversity analysis.

*Streptomycetes* were isolated from the soil samples using a serial dilution plating technique. A 10^−1^ dilution of the soil sample was prepared and vortexed for 10 min, followed by two subsequent tenfold dilutions to achieve 10^−2^ and 10^−3^ concentrations. Aliquots of 20 μL, 100 μL and 100 μL from 10^−1^, 10^−2^ and 10^−3^ dilutions, respectively, were plated onto Gauze’s medium No.1 plates. The plates were inverted and incubated at 30 °C for 3–5 days. A single colony was selected for purification based on their distinct morphological features in the plate. Colonies were obtained by employing the three-sector streaking method within the streak plate technique and followed those previously described culture conditions, which were finally preserved as glycerol suspensions (25%, *v*/*v*) at −80 °C with tryptic soy broth at 28 °C, 180 revolutions per minute (rpm) for 3–5 days.

### 2.2. 16S rRNA Gene and Phylogeny

For HC307^T^, cell culture was performed using tryptic soy broth, and a TIANamp bacterial DNA kit (TIANGEN company, Beijing, China) was used to obtain genomic DNA. PCR amplification of the 16S rRNA gene was performed using the universal primers 27F and 1492R [22]. The gene sequences with high similarity were selected using a BLAST-n (https://blast.ncbi.nlm.nih.gov/Blast.cgi) search of NCBI accessed on 13 July 2023. The gene phylogenetic tree was reconstructed with the Neighbor-Joining algorithms using MEGA 11 [23]. A bootstrap analysis with 1000 repetitions was conducted to assess the confidence of the branch nodes.

### 2.3. Genome Sequencing, Assembly and Annotation

The genomic sequencing of strain HC307^T^ was performed by Tsingke Biotech (Beijing Tsingke Biotechnology Co., Ltd., Xi’an, China) utilizing the third generation standard Oxford Nanopore Technologies (ONT) sequencing technology and the Nanopore sequencing platform. Whole-genome sequencing of strain HC307^T^ was performed through the standard Oxford Nanopore Technologies (ONT) protocol. The genomic DNA was initially extracted by the SDS method; its purity, concentration, and integrity were assessed through Nanodrop, Qubit and 0.35% agarose gel electrophoresis. The Ligation sequencing gDNA Kit (SQK-LSK109, ONT, London, UK) was used for library construction, including DNA damage repair, end repair, adapter ligation and library quantification. Then, the sequencing was carried out on the Nanopore sequencing platform. Following base calling, the sequencing data underwent further filtering to remove low-quality reads and segment fragments (<2000 bp).

The filtered reads were then assembled using Canu v1.5 software [24], and the assembly was subsequently corrected using Racon v3.4.3. Circularization and adjustment of the start site were performed via Circlator v1.5.5, resulting in a complete genome.

The obtained genome for each sample had a MeanQual of no less than 8, and a coverage of no less than 100× in Nanopore sequencing data. This exhibited high reliability and accuracy, ensuring robust genomic analyses and dependable findings.

Coding DNA sequence (CDS) regions were predicted with Prodigal v2.6.3. Repetitive elements were identified through comparative analysis of the assembled genome against known repeat databases using RepeatMasker v4.0.5. rRNA genes were predicted from the Rfam database, employing covariance models with Infernal v1.1.3. And tRNA genes were identified using tRNAscan-SE v2.0. The paralogous genes were selected using BLASTP, then compared with Non-Redundant protein (Nr), Gene Ontology (GO), Kyoto Encyclopedia of Genes and Genomes (KEGG), evolutionary genealogy of genes: Non-supervised Orthologous Groups (eggNOG), Carbohydrate Active Enzymes (CAZy), Comprehensive Antibiotic Resistance Database (CARD) and Pathogen-Host Interactions (PHI) database to complete the functional annotation of the protein sequences. Notably, GO annotation was further facilitated by the implementation of Blast2GO v2.5.

The closely related *Streptomyces* species were chosen by phylogenetic analysis inferring using 16S rRNA gene sequences. To further elucidate the pairwise phylogenetic relationships among the nearest strains, whole genome data were obtained, and 16S rRNA gene-based ANI value was calculated using EzBioCloud (www.ezbiocloud.net/tools/ani) [25] accessed on 17 October 2024. This initial analysis provided a rapid assessment of relatedness. For a more precise determination of genomic relatedness, dDDH value was predicted using the Genome-to-Genome Distance Calculator (http://ggdc.dsmz.de/ggdc.php) version 3.0 accessed on 18 October 2024 [26]. Genome sequences of type strains could be found in the Type Strain Genome Server (TYGS) database [27]. A robust genome-based phylogenetic tree was then constructed utilizing the Genome Blast Distance Phylogeny (GBDP) approach and MASH algorithms implemented in FASTME 2.1.6.1, with 100 bootstrap replicates to assess branch support.

### 2.4. Phenotypic Characteristics and Chemotaxonomy Test

A range of International Streptomyces Project (ISP) media was used to assess the cultural characteristics of strain HC307^T^, including ISP 1 (tryptone yeast extract broth), ISP 2 (yeast extract malt extract agar), ISP 3 (oat meal agar), ISP 4 (inorganic salt starch agar), Gauze’s medium No.1, Czapek-Dox agar, Bennett’s agar and potato dextrose agar [28] and repeated 3 times. Then, the plates were cultivated at 28 °C for 7 days. The morphological characterizations of the strain HC307^T^ were recorded and described, including the colors of mature sporophyte, spore pile, substrate hyphae, the colors and characteristics of aerial hyphae, substrate mycelium, soluble pigments as well as water absorption spot on these media, especially Gauze’s medium No.1. The hyphal morphology of strain HC307^T^ was observed using both Biological Microscope (BM, B302, OPTEC, Chongqing, China) and scanning electron microscopy (SEM, JSM-5600LV, JEOL, Akishima, Japan) [29]. The sample was prepared by the methodology described below. The growth characteristics of strain HC307^T^ were determined across a range of pH (3–12) in liquid Gauze’s medium No.1, and at various temperatures (25–40 °C) and NaCl concentrations (0–9%, *w*/*v*) on solid Gauze’s medium No.1 [30]. The strain was subjected to a battery of biochemical tests including proteolysis (gelatin, casein), carbohydrate utilization (starch, cellulose), lipolysis (tween hydrolysis) and carbon and nitrogen source utilization [31]. Polar lipid identification was conducted through two-dimensional thin-layer chromatography, following the procedures outlined by Jiuyan Xie [32]. Detection of total lipids, phospholipids, aminolipids and glycolipids were achieved through the application of molybdenum phosphoric acid, molybdate, ninhydrin and *α*-naphthol, respectively.

### 2.5. Secondary Metabolite Biosynthetic Gene Cluster Prediction

To further elucidate the potential biotechnological applications of strain HC307^T^, BGCs were predicted by anti-SMASH 7.1.0 [33]. This analysis aimed to investigate the diversity of its natural product repertoire, thereby enhancing the prediction of its applicative and research significance.

## 3. Results

All *Streptomycete* strains exhibited robust growth on Gauze’s medium No.1. There were thirty-three *Streptomycete* strains isolated from the medium, exhibiting irregular colony margins and a powdery sporulation on the colony surface.

### 3.1. 16S rRNA Gene and Phylogeny Analysis

**Figure 1:**
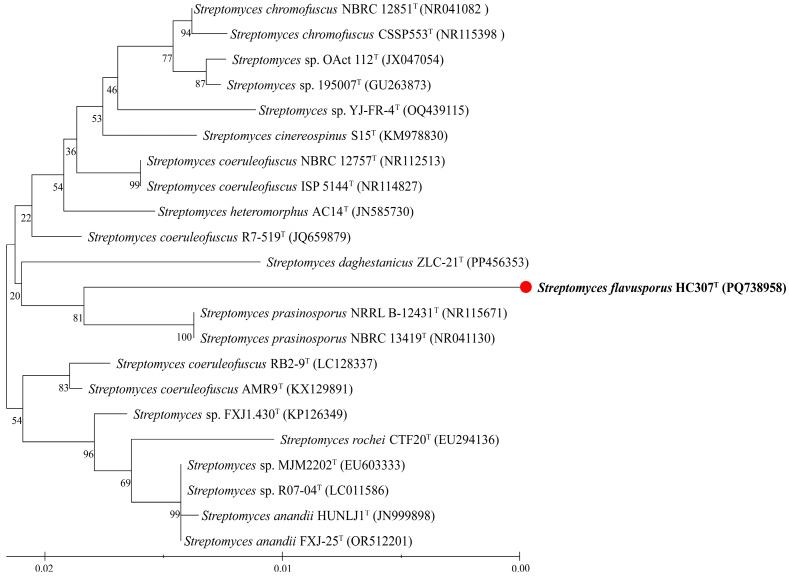
Neighbor-joining analysis of 16S rRNA gene sequences showing the phylogenetic relationship of strain HC307^T^ within the genus *Streptomyces*. Bootstrap values based on 1000 replicates are displayed at the branch nodes. The scale bar represents 0.02 substitutions per nucleotide. The strain HC307^T^ is marked in bold font and red circle, the superscript “T” indicates the type strain.

A 1411 bp 16S rRNA gene sequence was obtained from strain HC307^T^ and sequenced using the Sanger method, exhibiting the highest similarity to those of *Streptomyces prasinosporus* NRRL B-12431^T^ (97.5%), *Streptomyces prasinosporus* NBRC 13419^T^ (97.5%), *Streptomyces chromofuscus* NBRC 12851^T^ (97.3%), *Streptomyces coeruleofuscus* R7-519^T^ (97.3%) and *Streptomyces chromofuscus* DSM 40273^T^ (97.3%). Phylogenetic analysis of the 16S rRNA gene placed strain HC307^T^ within the genus *Streptomyces*; however, it formed a separate branch that originates from the same node as other *Streptomyces* species (Figure 1). The phylogenetic tree revealed a high degree of bootstrap value, indicating that the strain HC307^T^ was clearly located within the genus of *Streptomyces*.

### 3.2. Genome Features

The genome of strain HC307^T^ was assembled into 10.0 Mb and consisted of 2 Contigs (N50, 10,014,678 bp) after further filtering of reads for low quality and segment fragments (<2000 bp). The genomic DNA G+C content of strain HC307^T^ was 70.0 mol%. Strain HC307^T^ contained 18 rRNA and 78 tRNA genes in the non-coding RNA prediction, including 6 16S rRNA, 6 5S rRNA and 6 23S rRNA genes. The repetitive sequence was 141,214 bp in size, representing 1.41% of the genomic sequence. The draft genomic features of strains HC307^T^ are described in Table S1. Of 9153 genes for comparison and annotation in the Nr database, 7669 genes (83.8%) were matched to *Streptomyces* and 1484 (16.2%) were related to other species (Figure S1).

**Figure 2:**
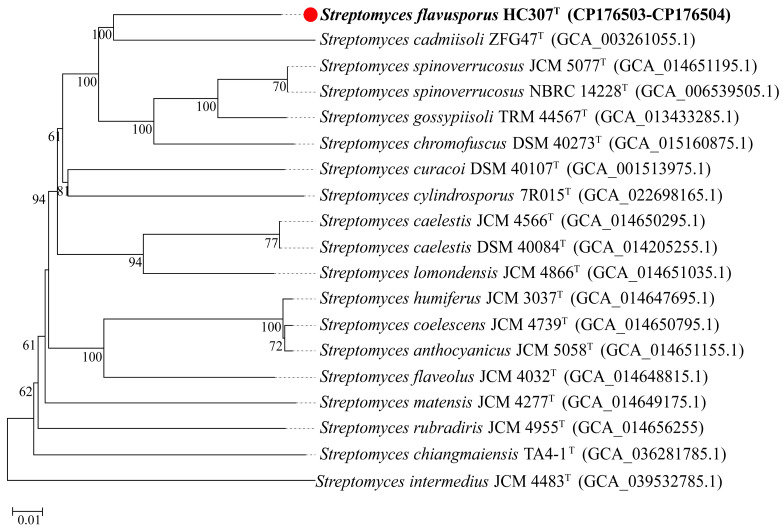
Whole-genome sequence analysis using the GBDP algorithm showing the phylogenetic classification of HC307^T^ within TYGS. Branch lengths are scaled according to the GBDP distance formula d_5_ [26]. The numbers above the branches represent the percentage of GBDP pseudo-bootstrap support values >60% from 100 replicates, with an average branch support of 82.3%. The scale bar represents 0.01 substitutions per nucleotide. The tree is rooted at the midpoint. The strain HC307^T^ is marked in bold font and red circle, the superscript “T” indicates the type strain.

In comparative analysis of closely related strains under TYGS, HC307^T^ exhibited ANI values of 86.9%, 87.2% and 86.6% with *S. cadmiisoli* ZFG47^T^, *S. gossypiisoli* TRM 44567^T^ and *S. chromofuscus* DSM 40273^T^, respectively. The corresponding dDDH values were 33.3%, 33.8% and 32.6%, while the GC content differences were 0.73%, 0.74% and 1.58%, respectively. Evidently, the confidence interval (CI) values of dDDH were 30.9–35.8%, 31.3–36.3% and 30.1–35.1%, respectively (Table S2). Employing the GBDP method and a tree-building service, a phylogenetic tree for strain HC307^T^ was constructed based on the whole-genome sequence (Figure 2).

### 3.3. Phenotypic Characteristics and Chemotaxonomy

Strain HC307^T^ was grown on a variety of agar plates for seven days, the aerial hyphae, substrate mycelium, diffusible pigment and growth were morphologically characterized. The results are shown in Table S3. Strain HC307^T^ exhibited moderate growth on ISP 1, ISP 3 and ISP 4, with robust growth on Gauze’s medium No.1 and Czapek-Dox agar. The pink pigment was produced on Gauze’s medium No.1. It grew weakly on Bennett’s agar and ISP 2, and basically did not grow on potato dextrose agar. On Gauze’s medium No.1, strain HC307^T^ formed tightly adherent colonies with a dry, powdery surface. Its aerial hyphae were light yellow, while the substrate mycelium appeared brown and exhibited the production of a pink pigment (Figure S2).

The well-developed hyphae of strain HC307^T^ were observed after culturing on Gauze’s medium No.1 for 5 days. The hyphae exhibited a radial growth pattern, extending outward from a central point. They were characterized by a linear, slightly curved morphology with abundant smooth, branched and a-septate aerial hyphae (Figure S3).

The strain could utilize NaCl concentrations ranging from 0% to 6%, with optimal growth at 0%. With increasing NaCl concentrations, it grew weaker and weaker until 7% NaCl concentration. The strain HC307^T^ was found to grow, ranging from 25 °C to 40 °C (optimum temperature 28 °C). With the increase in culture temperature and the extension of incubation time, strain HC307^T^ produced a pigment that resulted in a pink halo around the colony, which gradually deepened in color. It was found that strain HC307^T^ could grow within the pH range of 5–9 and had a good growth state within the pH range of 5–7. A detailed description of strain HC307^T^ is shown in Table 1.

The bacteria demonstrated a positive phenotype for urease, gelatinase, amylolytic and lipolytic activities, and a negative phenotype for production of hydrogen sulfide, indole and tryptophan. The strain exhibited a positive Voges-proskauer reaction, consistent with diacetyl fermentation, while a negative methyl red test indicated an inability to produce significant quantities of mixed acids from glucose fermentation.

The strain HC307^T^ could fully utilize D-trehalose, D-fibrodisaccharide, lactose, mannitol, arabinose, sucrose, maltose, glucose, inositol, *β*-cyclodextrin as carbon sources. The organism exhibited the ability to utilize a wide range of common carbohydrates as carbon sources. The results of nitrogen source utilization showed that the strain HC307^T^ could use a wide range of nitrogen sources, including proline, ammonium nitrate, methionine, histidine, glycine, tyrosine and ammonium salts such as ammonium phosphate dibasic. Strain HC307^T^ exhibited detailed physiological tests characterization, and the results are documented in Table 2.

The polar lipid profile of strain HC307^T^ comprises diphosphatidylglycerol (DPG), phosphatidylglycerol (PG), phosphatidylethanolamine (PE), alongside unidentified aminophospholipids (UAPL1-3), phospholipids (UPL1-3), an aminolipid (UAL) and lipids (UL1-3). The details are shown in Figure S4.

We compared phenotypic characteristics of strain HC307^T^ and type strains of *S. prasinosporus*, *S. cadmiisoli*, *S. gossypiisoli* and *S. chromofuscus* for pH and salt tolerance, polar lipid composition and utilization of selected carbon sources, and found that strain HC307^T^ exhibited distinct phenotypic characteristics from these other *Streptomyces* type strains (Table 3).

### 3.4. Characteristics of Secondary Metabolite BGCs

The genome of strain HC307^T^ revealed 32 secondary metabolite synthesis related gene clusters using antiSMASH 7.1.0, of which 13 showed similarity exceeding 60% to known BGCs. Among them, eight BGCs were completely like known gene clusters. Their predicted products included albaflavenone, *ε*-poly-L-lysine and neocarzilin A/B. These BGCs are shown fully in Table S4.

## 4. Discussion

*Streptomycetes* are known for their remarkable ecological diversity, and their extensive exploration has been a focal point of research by numerous scholars. Like daptomycin [37], a cyclic lipopeptide antibiotic, derived from *Streptomyces roseosporus*, exerted its bactericidal activity by disrupting membrane curvature, leading to ion leakage and the dissipation of membrane potential. This disruption subsequently inhibited critical cellular processes, including protein, DNA and RNA synthesis, ultimately inducing bacterial cell death. In the United States, it had been approved for the treatment of complicated skin and skin structure infections (cSSSI). A researcher [38] had conducted research by comparing the taxonomic characteristics of a *Stretomyces* strain isolated from an extreme environment with those of a known *Streptomyces* of the same species. They found that the gene identification of the two was completely consistent. Nevertheless, analysis of the secondary metabolites indicated the presence of unknown compounds, potentially with a peptide structure, and further unique metabolites were isolated [38]. It suggested that extreme environments can stimulate the activation of silent gene clusters in *Streptomycete*, leading to the production of previously unknown compounds or antibiotics. In this study, a strain of *Streptomyces* exhibiting multiple enzyme-producing capabilities and the potential to synthesize diverse secondary metabolites was isolated from an extreme environment. The study described the taxonomic status of the strain by integrating genetic and phenotypic analysis. Additionally, the potential secondary metabolites of the strain were thoroughly analyzed using the antiSMASH prediction tool.

The 16S rRNA gene sequence between *S. prasinosporus* NRRL B-12431^T^, *S. chromofuscus* NBRC 12851^T^ and *S. coeruleofuscus* R7-519^T^ with low similarity (97.3–97.5%), which were significantly below the threshold (98.7%) [39,40,41] commonly used for species delineation. Furthermore, the strain formed a separate branch that originated from the same node as other *Streptomyces* species (Figure 1). The phylogenetic tree showed a high degree of bootstrap value, indicating that the strain HC307^T^ was clearly located within the genus of *Streptomyces*.

The whole genome of strain HC307^T^ was 10.0 Mb, and the GC content was 70.0 mol%, which was consistent with the characteristic high GC content typically observed in actinomycetes [4]. The G+C content differences within-species were almost exclusively below 1% [42]. So, the GC content difference between *S. prasinosporus* JCM 4816^T^ and *S. prasinosporus* NBRC 13419 ^T^ were below 1%. The whole genome of *S. prasinosporus* JCM 4816 ^T^ was 15.0 Mb in size, with a GC content of 72.5 mol% [43]. Compared to HC307^T^, the G+C content difference in *S. prasinosporus* JCM 4816 ^T^ completely showed low similarity (<10^−6^) [26,42], it could be inferred that the two strains exhibited a distant phylogenetic relationship. Furthermore, genomic analyses were conducted to determine ANI and dDDH values. The dDDH values between strain HC307^T^ and closely related strains ranged from 22.6% to 33.6%. These values were substantially lower than the threshold (70%) [41,44] typically used for species delineation. Additionally, the ANI values distributed between 78.3% and 87.5%, which were substantially below the 95% threshold commonly employed for species demarcation.

The Nr protein database consolidated protein sequence data from diverse sources, undergoing redundancy reduction to establish a comprehensive foundation for homology analyses, a principle where non-redundant protein sequences identifiable across multiple RefSeq genomes reflect taxonomic relationships [45]. Homology analysis of protein sequences indicated an 83.8% probability of strain HC307^T^ belonging to the genus *Streptomyces*. The collective genomic evidence strongly supported that strain HC307^T^ represented a novel species within the genus *Streptomyces*.

Compared to the similar type strains, including *S. prasinosporus*, *S. cadmiisoli*, *S*. *chromofuscus* and *S*. *gossypiisoli*, there were numerous phenotypic differences. Strain HC307^T^ demonstrated a broad range of nutritional versatility across various culture media. In contrast to *S. prasinosporus*, HC307^T^ exhibited limited growth on Bennett’s agar and failed to proliferate on potato dextrose agar. Furthermore, it could produce a pink pigment in Gauze’s medium No.1, a characteristic observed under specific culture conditions and potentially related to its efficient utilization of carbon and nitrogen sources, as well as the temperature and duration [46].

Conversely, *S. prasinosporus* displayed light to moderate growth on Bennett’s agar, whereas *S. chromofuscus* showed good growth on the same medium. Moreover, the strain could utilize NaCl tolerance ranging from 0% to 6%, with optimal growth at 0%, while *S. cadmiisoli* tolerated concentrations between 0 and 12%. The clear differences in salt tolerance observed between the two strains strongly suggested they represented distinct species. Strain HC307^T^ exhibited a broad substrate utilization profile for carbon and nitrogen sources, demonstrating the ability to utilize a wide range of carbon substrates with the exception of D-fructose, xylan, D-sorbitol and sodium acetate, while the *S. prasinosporus* could only selectively utilize glucose, arabinose and fructose [28]. Antibiotic formation was predicated on the metabolic processing of carbon and nitrogen substrates [47]. Specifically, carbon could provide phosphorylated sugars through metabolic pathways such as glycolysis and the pentose phosphate pathway, which served as the foundation for biosynthetic pathways of secondary metabolites. In primary metabolism, nitrogenous compounds, including amino acids and nitro-compounds, contribute to antibiotic biosynthesis. Therefore, supplementation with nitrogenous compounds facilitated antibiotic biosynthesis [48]. Furthermore, biochemical analysis revealed a diverse enzymatic profile for strain HC307^T^. It demonstrated the ability to secrete a range of extracellular enzymes, including amylase, urease, esterase and gelatinase, but lacked the capacity to produce melanin and H_2_S.

Differences in polar lipid composition, a major constituent of microbial cell membranes, suggested genetic and physiological divergences among species. The polar lipid profile of strain HC307^T^ comprised DPG, PG, PE and UAPL1-3, UPL1-3, UAL, UL1-3. This observation is consistent with the established polar lipid profile of *Streptomyces* species, which typically comprised PG, PE and cardiolipin (CL) [49]. Importantly, the polar lipid composition of *S. gossypiisoli* included PE, PG, phosphatidylinositol (PI), phosphatidyl-choline (PC) and phosphatidylinositol mannoside (PIM). The differences between the two strains suggested that they represent separate species. Based on genotypic and phenotypic data, strain HC307^T^ is proposed as a novel species within the genus *Streptomyces*.

Prediction of the strain’s BGCs revealed eight clusters exhibiting complete similarity to established gene clusters, including those encoding bioactive metabolite production. Cluster 1.1 had 47 genes and 100% similarity with the gene cluster for Type III polyketide synthase (T3PKS). The predicted product was alkylresorcinol (ARs) which played a structural role in the membranes of producing organisms and had properties such as antioxidant, antibacterial and anticytotoxic activities [50,51]. ARs modulated membrane viscosity through interactions with lipid molecules and macromolecules, consequently attenuating their functional activities. It mediated their anticancer effects by targeting multiple sites within tumor cell DNA, resulting in DNA fragmentation and the disruption of DNA repair pathways, consequently suppressing neoplastic cell proliferation [52].

Among the above 32 BGCs, there were two nonribosomal peptide synthetases (NRPSs), three Type I polyketide synthases (T1PKSs), two Type II polyketide synthases (T2PKSs), two T3PKSs and other BGCs, such as oleanders, iron carriers, ectoine, NAPAA, and so on. As is known that the polyketide synthase (PKS) gene cluster and NRPS genes had the potential to synthesize antimicrobial drugs, more than 23,000 PKS and NRPS natural products had been identified and characterized, including antibiotics and drugs with antitumor, anthelmintic, immunosuppressive properties and other biological activities [5,7,8,53,54].

In other BGCs, there were also predictions of antibiotic-related metabolites. Neocarzilin A was a class of natural products containing trichloromethyl ketone and could be served as an effective inhibitor for targeting the VAT-1 pathway to control cancer cell movement [55]. Cluster 1.13 was analyzed and found that the main biological amino acids were ScbA/BarX family with 94.4% similarity, which was hypothesized to have the potential to synthesize *γ*-butyrolactones [56]. As hormone-like signaling molecules, *γ*-butyrolactones regulated diverse physiological processes, particularly the induction of antibiotic production [57]. Ectoine was a natural osmotic substance produced by extremophiles, playing a crucial role in the survival of bacteria in hypertonic environments [58]. Aspartate served as the precursor for ectoine biosynthesis, where L-aspartate kinase (Ask) and NADPH-dependent L-aspartate-*β*-semialdehyde dehydrogenase (Asd) function in the production of L-aspartate-*β*-semialdehyde (ASA), which were subsequently transformed into ectoine by the catalytic action of EctA, EctC, and EctB [59]. The cell was therefore a potential source of biologically active secondary metabolites.

## 5. Conclusions

A strain of *Streptomyces* was isolated from a soil sample collected in Xizang (Tibet), China. Genomic and phenotypic analyses in this study have demonstrated notable differences between strain HC307^T^ and its closely related species, evidenced by disparities in ANI and dDDH values, physiological traits and polar lipid profiles, affirming its status as a novel species. In addition, the strain demonstrates versatile enzymatic capabilities, and when coupled with the characterization of its secondary metabolite BGCs, reveals significant potential for generating structurally diverse secondary metabolites with potential bioactivities.

In conclusion, the present findings contribute to the expansion of *Streptomyces* bioresources and support the unearthing of novel secondary metabolites. Future studies will focus on the BGCs of the strain, particularly the activation of silent clusters. By integrating synthetic biology approaches with metabolomics analyses, we aim to explore its potential metabolic diversity and isolate active compounds with promising applications.

### Description of Streptomyces flavusporus *sp. nov.*

*Streptomyces flavusporus* (fla.vu.spo’rus. L. masc. adj. *flavus*, yellow, describing the yellow substrate mycelium; Gr. fem. n. *spora*, a spore; N.L. masc. adj. *flavusporus*, yellow-spored).

Cells are Gram-stain-positive, the colony is yellow in color, dry and powdery when cultured in Gauze’s medium No.1 for 7 days. With prolonged incubation, it can release pink soluble pigment, giving the colonies a tangerine color. Cells can grow well in Czapek-Dox agar, Gauze’s medium No.1, ISP 1, ISP 3 and ISP 4, grow weekly in Bennett’s agar and ISP 2, and cannot grow in potato dextrose agar. The growth temperature range of the strain is 25–40 °C, with an optimum at 28 °C on Gauze’s medium No.1. In the same medium, bacteria can grow within pH 5.0–9.0 (optimum, pH 5.0–7.0) and 0–6% NaCl (optimum, 0% NaCl). The strain exhibits positive results for gelatin, Tween-20, Tween-80, starch, casein, and urea hydrolysis but negative results for cellulose and tyrosine hydrolysis. Additionally, nitrate reduction and indole production are negative. The strain is unable to utilize xylan, D-fructose, D-sorbitol and sodium acetate as sole carbon sources. Similarly, arginine, cysteine, phenylalanine, glutamic acid, diammonium hydrogen phosphate, ammonium acetate and ammonium molybdate cannot serve as sole nitrogen sources. The polar lipid profile contains DPG, PG, PE, UAPL1-3, UPL1-3, UAL and UL1-3.

The strain, HC307^T^ (=DSM 35222^T^=CGMCC 32047^T^), was isolated from a soil sample from Naidong District, Shannan City, Xizang (Tibet), China. The strain exhibited a GC content of 70.0 mol%. The 16S rRNA gene sequence and draft genome sequence of this strain had been deposited in the NCBI GenBank database under accession numbers PQ738958 and CP176503-CP176504, respectively.

## Figures and Tables

**Table 1:** Morphological and physiological characteristics of strain HC307^T^ in Gauze’s medium No.1.

Morphological Characterizations					
Gause’s No.1 medium		Aerial mycelium		Substrate mycelium	
Color		Yellow		Brown	
Colony morphology		Radial			
Elevation		Raised			
Texture		Dry, powdery			
Pigmentation		Pink			
Physiological characterizations					
Range of NaCl tolerance			0–6% (optimum, 0% NaCl)		
pH growth range			Temperature growth range		
pH 3	-		25 °C		+
pH 4	-		28 °C		++
pH 5	++		30 °C		+
pH 6	++		34 °C		+
pH 7	++		37 °C		+
pH 8	+		40 °C		+
pH 9	+				
pH 10	-				
pH 11	-				
pH 12	-				

++, optimal growth; +, normal growth; -, no growth.

**Table 2:** Physiological and biochemical characteristics of strain HC307^T^.

Characteristic	Result	Characteristic	Result	Characteristic	Result
Biochemical Test		Nitrogen Source Utilization		Carbon Source Utilization	
Nitrate reduction	-	Proline	+	D-trehalose	+
Starch hydrolysis	+	Ammonium nitrate	+	Xylan	-
Gelatin liquefaction	+	Methionine	+	D-fibrodisaccharide	+
Voges-proskauer	+	Cysteine	-	Sodium acetate	-
MR	-	Ammonium acetate	-	D-fructose	-
Indole	-	Ammonium molybdate	-	D-sorbitol	-
Hydrogen sulfide production	-	Ammonium phosphate dibasic	+	Lactose	+
Cellulose hydrolysis	-	Phenylalanine	-	Mannitol	+
Proteolysis	+	Histidine	+	Arabinose	+
Hydrolysis of tween-20	+	Arginine	-	Sucrose	+
Hydrolysis of tween-80	+	Ammonium dihydrogen phosphate	-	Maltose	+
Urea hydrolysis	+	Glycine	+	Glucose	+
		Tyrosine	+	Inositol	+
		Glutamic acid	-	*β*-cyclodextrin	+

+, optimal growth; -, no growth.

**Table 3:** Comparison of phenotypic characteristics of strain HC307^T^ and related type strains.

Characteristic	Type Strain				
	1	2	3	4	5
NaCl (*w*/*v*, %)	0–6	2–5	0–12	0–7	2–5
pH (Optimum)	5–7	ND	7.0	7–8	ND
Diphosphatidylglycerol	+	ND	+	+	ND
Phosphatidylethanolamine	+	ND	+	+	ND
Phosphatidylglycerol	+	ND	+	-	ND
Phosphatidylcholine	-	ND	-	+	ND
Phosphatidylinositol	-	ND	-	+	ND
Phosphatidylinositol mannoside	-	ND	-	+	ND
Glucose	+	+	-	ND	+
Xylan	-	-	-	-	+
Inositol	+	-	ND	ND	+
Mannitol	+	+	-	+	+

1, Strain HC307^T^; 2, *S. prasinosporus* 40506^T^ [28]; 3, *S. cadmiisoli* ZFG47^T^ [34]; 4, *S. gossypiisoli* TRM 44567^T^ [35]; 5, *S. chromofuscus* DSM 40273^T^ [36]; ND, no data available; +, optimal growth; -, negative.

## Data Availability

The 16S rRNA gene sequence and draft genome sequence of the strain are publicly available in the NCBI GenBank database under accession numbers PQ738958 and CP176503-CP176504, respectively.

## Supplementary Materials

The following supporting information can be downloaded at: https://www.mdpi.com/article/10.3390/microorganisms13051001/s1. Figure S1: Statistical map of Nr annotated species of strain HC307^T^; Figure S2: The colony morphology of strain HC307^T^ on eight mediums after being incubated at 28 °C for 7 days; (a) Gauze’s medium No.1; (b) Czapek-Dox agar; (c) ISP 1; (d) ISP 2; (e) ISP 3; (f) ISP 4; (g) Bennett’s agar; (h) Potato dextrose agar; Figure S3: The morphology of hyphae of strain HC307^T^; (a) hyphae of HC307^T^ observed under 400-fold optical microscopy; (b) hyphae of HC307^T^ observed under scanning electron microscopy; Figure S4: The polar lipid profile of strain HC307^T^ after separation by two-dimensional TLC; (a) Phosphomolybdic acid staining; (b) Molybdate staining; (c) Ninhydrin staining; (d) *α*-naphthol staining; DPG, diphosphatidyl glycerol; PE, phospatidylethanolamine; PG, phosphatidyl glycerol; UAPL1-3, unidentified aminophospholipids; UPL1-3, unidentified phospholipids; UAL, unidentified aminolipid; UL1-3, unidentified lipids; Table S1: The draft genomic features of strain HC307^T^ are described; Table S2: Pairwise comparisons of the genome of strain HC307^T^ (PQ738958) versus type strain genomes with TYGS (https://tygs.dsmz.de/) accessed on 18 October 2024 and ANI (www.ezbiocloud.net/tools/ani) accessed on 17 October 2024; Table S3: Culture characteristics of the strain HC307^T^ in 28 °C for 7 days; Table S4: The analysis of biosynthetic pathways in strain HC307^T^ by antiSMASH 7.1.0.

## Abbreviations

BGCs, biosynthetic gene clusters; ANI, average nucleotide identity; GBDP, genome blast distance phylogeny; dDDH, digital DNA-DNA hybridization; PKS, polyketide synthase; NRPS, nonribosomal peptide synthetase; T1PKS, Type I polyketide synthase; T2PKS, Type II polyketide synthase; T3PKS, Type III polyketide synthase; TYGS, type strain genome server; GGDC, genome-to-genome distance calculator, ISP, international Streptomyces project medium.

## References

[1] Hasani A., Kariminik A., Issazadeh K. (2014). *Streptomycetes*: Characteristics and their antimicrobial activities. Int. J. Adv. Biol. Biomed..

[2] Kämpfer P., Dworkin M., Falkow S., Rosenberg E., Schleifer K.H. (2006). The family *Streptomycetaceae*, Part I: Taxonomy. The Prokaryotes.

[3] Selim M.S.M., Abdelhamid S.A., Mohamed S.S. (2021). Secondary metabolites and biodiversity of actinomycetes. J. Genet. Eng. Biotechnol..

[4] Barka E.A., Vatsa P., Sanchez L., Gaveau-Vaillant N., Jacquard C., Meier-Kolthoff J.P., Klenk H.P., Clément C., Ouhdouch Y., Van Wezel G.P. (2016). Taxonomy, physiology, and natural products of actinobacteria. Microbiol. Mol. Biol. Rev..

[5] Jagannathan S.V., Manemann E.M., Rowe S.E., Callender M.C., Soto W. (2021). Marine actinomycetes, new sources of biotechnological products. Mar. Drugs.

[6] Parte A.C., Sardà Carbasse J., Meier-Kolthoff J.P., Reimer L.C., Göker M. (2020). List of prokaryotic names with standing in nomenclature (LPSN) moves to the DSMZ. Int. J. Syst. Evol. Microbiol..

[7] Quinn G.A., Banat A.M., Abdelhameed A.M., Banat I.M. (2020). *Streptomyces* from traditional medicine: Sources of new innovations in antibiotic discovery. J. Med. Microbiol..

[8] Alam K., Mazumder A., Sikdar S., Zhao Y.M., Hao J., Song C., Wang Y., Sarkar R., Islam S., Zhang Y. (2022). *Streptomyces*: The biofactory of secondary metabolites. Front. Microbiol..

[9] Chen Y.Y., Chen L.Y., Chen P.J., Shazly M.E., Peng B.R., Chen Y.C., Su C.H., Su J.H., Sung P.J., Yen P.T. (2022). Probing anti-leukemic metabolites from marine-derived *Streptomyces* sp. LY1209. Metabolites.

[10] Hassan H.M., Degen D., Jang K.H., Ebright R.H., Fenical W. (2015). Salinamide F, new depsipeptide antibiotic and inhibitor of bacterial RNA polymerase from a marine-derived *Streptomyces* sp.. J. Antibiot..

[11] Kim L. (2020). The science of antibiotic discovery. Cell..

[12] Van Bergeijk D.A., Terlouw B.R., Medema M.H., Van Wezel G.P. (2020). Ecology and genomics of actinobacteria: New concepts for natural product discovery. Nat. Rev. Microbiol..

[13] Wright G.D. (2014). Something old, something new: Revisiting natural products in antibiotic drug discovery. Can. J. Microbiol..

[14] Trenozhnikova L.P., Baimakhanova G.B., Baimakhanova B.B., Balgimbayeva A.S., Daugaliyeva S.T., Faizulina E.R., Tatarkina L.G., Spankulova G.A., Berillo D.A., Beutler J.A. (2024). Beyond traditional screening: Unveiling antibiotic potentials of actinomycetes in extreme environments. Heliyon.

[15] Sunaryanto R., Marwoto B., Hartoto L., Mas’ud Z.A., Irawadi T. (2011). Cyclo (tyrosyl-prolyl) produced by sp.: Bioactivity and molecular structure elucidation *Streptomyces*. Microbiol. Indones.

[16] Alshaibani M.M., Jalil J., Sidik N.M., Edrada-Ebel R., Zin N.M. (2016). Isolation and characterization of cyclo-(tryptophanyl-prolyl) and chloramphenicol from *Streptomyces* sp. SUK 25 with antimethicillin-resistant *Staphylococcus aureus* activity. Drug Des. Dev. Ther..

[17] Jose P.A., Jebakumar S.R.D. (2014). Unexplored hypersaline habitats are sources of novel actinomycetes. Front. Microbiol..

[18] Abdelkader M.S.A., Philippon T., Asenjo J.A., Bull A.T., Goodfellow M., Ebel R., Jaspars M., Rateb M.E. (2018). Asenjonamides A-C, antibacterial metabolites isolated from *Streptomyces asenjonii* strain KNN 42.f from an extreme-hyper arid Atacama Desert soil. J. Antibiot..

[19] Baral B., Akhgari A., Metsä-Ketelä M. (2018). Activation of microbial secondary metabolic pathways: Avenues and challenges. Synth. Syst. Biotechnol..

[20] Hei Y., Zhang H., Tan N., Zhou Y., Wei X., Hu C., Liu Y., Wang L., Qi J., Gao J.M. (2021). Antimicrobial activity and biosynthetic potential of cultivable actinomycetes associated with Lichen symbiosis from Qinghai-Tibet Plateau. Microbiol. Res..

[21] Ser H.L., Zainal N., Palanisamy U.D., Goh B.H., Yin W.F., Chan K.G., Lee L.H. (2015). *Streptomyces gilvigriseus* sp. nov., a novel actinobacterium isolated from mangrove forest soil. Antonie Van Leeuwenhoek..

[22] Huq M.A., NAM K., Rahman M.S., Rahman M.M., Parvez M.A.K., Kang K.K., Akter S. (2024). *Nocardioides agri* sp. nov., isolated from garden soil. Int. J. Syst. Evol. Microbiol..

[23] Tamura K., Stecher G., Kumar S. (2021). MEGA11: Molecular evolutionary genetics analysis version 11. Mol. Biol. Evol..

[24] Koren S., Walenz B.P., Berlin K., Miller J.R., Bergman N.H., Phillippy A.M. (2017). Canu: Scalable and accurate long-read assembly via adaptive k-mer weighting and repeat separation. Genome Res..

[25] Alanjary M., Steinke K., Ziemert N. (2019). AutoMLST: An automated web server for generating multi-locus species trees highlighting natural product potential. Nucleic Acids Res..

[26] Meier-Kolthoff J.P., Auch A.F., Klenk H.P., Göker M. (2013). Genome sequence-based species delimitation with confidence intervals and improved distance functions. BMC Bioinf..

[27] Kolthoff J.P.M., Göker M. (2019). TYGS is an automated high-throughput platform for state-of-the-art genome-based taxonomy. Nat. Commun..

[28] Tresner H.D., Hayes J.A., Backus E.J. (1966). *Streptomyces prasinosporus* sp. nov. a new green-spored species. Int. J. Syst. Evol. Microbiol..

[29] Chakraborty B., Kumar R.S., Almansour A.I., Perumal K., Nayaka S., Brindhadevi K. (2022). *Streptomyces filamentosus* strain KS17 isolated from microbiologically unexplored marine ecosystems exhibited a broad spectrum of antimicrobial activity against human pathogens. Process Biochem..

[30] Sandoval-Powers M., Králová S., Nguyen G.-S., Fawwal D.V., Degnes K., Lewin A.S., Klinkenberg G., Wentzel A., Liles M.R. (2021). *Streptomyces poriferorum* sp. nov., a novel marine sponge-derived actinobacteria species expressing anti-MRSA activity. Syst. Appl. Microbiol..

[31] Han D., Wang L., Luo Y. (2018). Isolation, identification, and the growth promoting effects of two antagonistic actinomycete strains from the rhizosphere of *Mikania micrantha* Kunth. Microbiol. Res..

[32] Xie J., Cheng K., Zhao D., Yang G., Qiao Z., Qiu S., Yu X., Liu H., Li T., Feng H. (2020). *Bacillus aquiflavi* sp. nov., isolated from yellow water of strongly flavored Chinese baijiu. Int. J. Syst. Evol. Microbiol..

[33] Blin K., Shaw S., Augustijn H.E., Reitz Z.L., Biermann F., Alanjary M., Fetter A., Terlouw B.R., Metcalf W.W., Helfrich E.J.N. (2023). antiSMASH 7.0: New and improved predictions for detection, regulation, chemical structures and visualisation. Nucleic Acids Res..

[34] Li K., Tang X., Zhao J., Guo Y., Tang Y., Gao J. (2019). *Streptomyces cadmiisoli* sp. nov., a novel actinomycete isolated from cadmium-contaminated soil. Int. J. Syst. Evol. Microbiol..

[35] Zhang Q.Y., Qin S., Luo X.X., Xia Z.F. (2021). *Streptomyces gossypiisoli* sp. nov., isolated from cotton soil in Xinjiang, PR China. Int. J. Syst. Evol. Microbiol..

[36] Wink J., Schumann P., Atasayar E., Klenk H.P., Zaburannyi N., Westermann M., Martin K., Glaeser S., Kämpfer P. (2017). *“Streptomyces caelicus*” an antibiotic producing species of the genus *Streptomyces* and *Streptomyces canchipurensis* Li et al. 2015 are later heterotypic synonym of *Streptomyces muensis* Ningthoujam et al. 2014. Int. J. Syst. Evol. Microbiol..

[37] Steenbergen J.N., Alder J., Thorne G.M., Tally F.P. (2005). Daptomycin: A lipopeptide antibiotic for the treatment of serious gram-positive infections. J. Antimicrob. Chemother..

[38] Sottorff I., Wiese J., Lipfert M., Preußke N., Sönnichsen F.D., Imhoff J.F. (2019). Different secondary metabolite profiles of phylogenetically almost identical *Streptomyces griseus* strains originating from geographically remote locations. Microorganisms.

[39] Kanchanasin P., Sripreechasak P., Suriyachadkun C., Rueangsawang K., Tanasupawat S., Phongsopitanun W. (2023). *Streptomyces cylindrosporus* sp. nov. and *Streptomyces spinosisporus* sp. nov.: Two new endophytic actinobacteria isolated from the roots of Barleria lupulina Lindl. Int. J. Syst. Evol. Microbiol..

[40] Kim M., Oh H.S., Park S.C., Chun J. (2014). Towards a taxonomic coherence between average nucleotide identity and 16S rRNA gene sequence similarity for species demarcation of prokaryotes. Int. J. Syst. Evol. Microbiol..

[41] Chun J., Oren A., Ventosa A., Christensen H., Arahal D.R., Costa M.S.d., Rooney A.P., Yi H., Xu X.W., Meyer S.D. (2018). Proposed minimal standards for the use of genome data for the taxonomy of prokaryotes. Int. J. Syst. Evol. Microbiol..

[42] Kolthoff J.P.M., Klenk H.P., Göker M. (2014). Taxonomic use of DNA G+C content and DNA-DNA hybridization in the genomic age. Int. J. Syst. Evol. Microbiol..

[43] Gonzalez-Silva A., Juan-Mendo M.S., Delgado-Prudencio G., Hernández-García J.A., Larios-Serrato V., Aguilar C., Villa-Tanaca L., Hernández-Rodríguez C. (2024). Comparative genomics and biosynthetic cluster analysis of antifungal secondary metabolites of three strains of *Streptomyces albidoflavus* isolated from rhizospheric soils. Microorganisms.

[44] Riesco R., Trujillo M.E. (2024). Update on the proposed minimal standards for the use of genome data for the taxonomy of prokaryotes. Int. J. Syst. Evol. Microbiol..

[45] Pavlopoulos G.A., Baltoumas F.A., Liu S., Selvitopi O., Camargo A.P., Nayfach S., Azad A., Roux S., Call L., Ivanova N.N. (2023). Unraveling the functional dark matter through global metagenomics. Nature.

[46] Abraham J., Chauhan R. (2017). Profiling of red pigment produced by *Streptomyces* sp. JAR6 and its bioactivity. 3 Biotech..

[47] Rammali S., Rahim A., Aalaoui M.E., Bencharki B., Dari K., Habach A., Abdeslam L., Khattabi A. (2024). Antimicrobial potential of *Streptomyces coeruleofuscus* SCJ isolated from microbiologically unexplored garden soil in Northwest Morocco. Sci. Rep..

[48] Krysenko S., Wohlleben W. (2024). Role of carbon, nitrogen, phosphate and sulfur metabolism in secondary metabolism precursor supply in *Streptomyces* spp.. Microorganisms.

[49] Sujarit K., Kudo T., Ohkuma M., Pathom-Aree W., Lumyong S. (2016). *Streptomyces palmae* sp. nov., isolated from oil palm (Elaeis guineensis) rhizosphere soil. Int. J. Syst. Evol. Microbiol..

[50] Martins T.P., Rouger C., Glasser N.R., Freitas S., Fraissinette N.B., Balskus E.P., Tasdemir D., Leao P.N. (2019). Chemistry, bioactivity and biosynthesis of cyanobacterial alkylresorcinols. Nat. Prod. Rep..

[51] Funabashi M., Funa N., Horinouchi S. (2008). Phenolic lipids synthesized by type III polyketide synthase confer penicillin resistance on *Streptomyces griseus*. J. Biol. Chem..

[52] Zabolotneva A.A., Shatova O.P., Sadova A.A., Shestopalov A.V., Roumiantsev S.A. (2022). An overview of alkylresorcinols biological properties and effects. J. Nutr. Metab..

[53] Chen L., Wang X.N., Bi H.Y., Wang G.Y. (2022). Antimicrobial biosynthetic potential and phylogenetic analysis of culturable bacteria associated with the sponge *ophlitaspongia* sp. from the Yellow Sea, China. Mar. Drugs.

[54] Laskaris P., Karagouni A.D. (2021). *Streptomyces*, greek habitats and novel pharmaceuticals: A promising challenge. Microbiol. Res..

[55] Gleissner C.M.L., Pyka C.L., Heydenreuter W., Gronauer T.F., Atzberger C., Korotkov V.S., Cheng W., Hacker S.M., Vollmar A.M., Braig S. (2019). Neocarzilin A is a potent inhibitor of cancer cell motility targeting VAT-1 controlled pathways. ACS Cent. Sci..

[56] Takano E. (2006). *γ*-Butyrolactones: *Streptomyces* signaling molecules regulating antibiotic production and differentiation. Curr. Opin. Microbiol..

[57] Kudo Y., Awakawa T., Du Y.L., Jordan P.A., Creamer K.E., Jensen P.R., Linington R.G., Ryan K.S., Moore B.S. (2020). Expansion of gamma-butyrolactone signaling molecule biosynthesis to phosphotriester natural products. ACS Chem. Biol..

[58] Sedeek A.M., Salah I., Kamel H.L., Soltan M.A., Nour E., Alshammar A., Rajoka M.S.R., Elsayed T.R. (2023). Genome-based analysis of the potential bioactivity of the terrestrial *Streptomyces vinaceusdrappus* strain AC-40. Biology.

[59] Ma Z., Wu C., Zhu L., Chang R., Ma W., Deng Y., Chen X. (2022). Bioactivity profiling of the extremolyte ectoine as a promising protectant and its heterologous production. 3 Biotech..

