# Supplementary material for: Streptomyces flavusporus sp. nov., a Novel Actinomycete Isolated from Naidong, Xizang (Tibet), China
Source: Microorganisms. 2025 Apr 27;13(5):1001. doi: 10.3390/microorganisms13051001 (PMC12113709; doi:10.3390/microorganisms13051001)
Supplement: Supplementary file 1 [file microorganisms-13-01001-s001.zip › microorganisms-3534452-supplementary.pdf]

Supplementary Material

Streptomyces flavusporus sp. nov., a Novel Actinomycete Isolated from Naidong, Xizang (Tibet), China

Dan Tang *, Xiaoxia Zhou, Haolin Qian, Yu Jiao and Yonggang Wang
School of Life Science and Engineering, Lanzhou University of Technology, Lanzhou, Gansu, 730050, China
* Correspondence: tang_danny125@163.com

1. Supplementary Figures

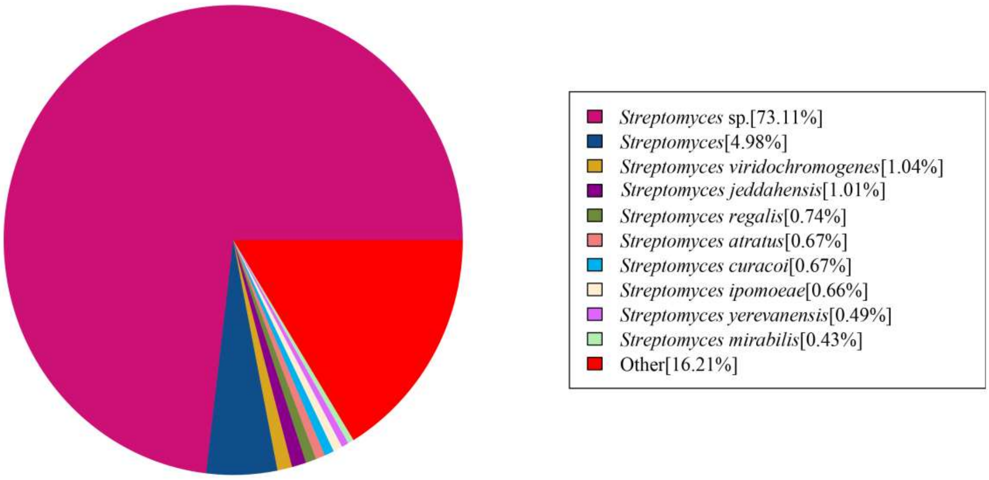

Figure S1. Statistical map of Nr annotated species of strain HC307^T.

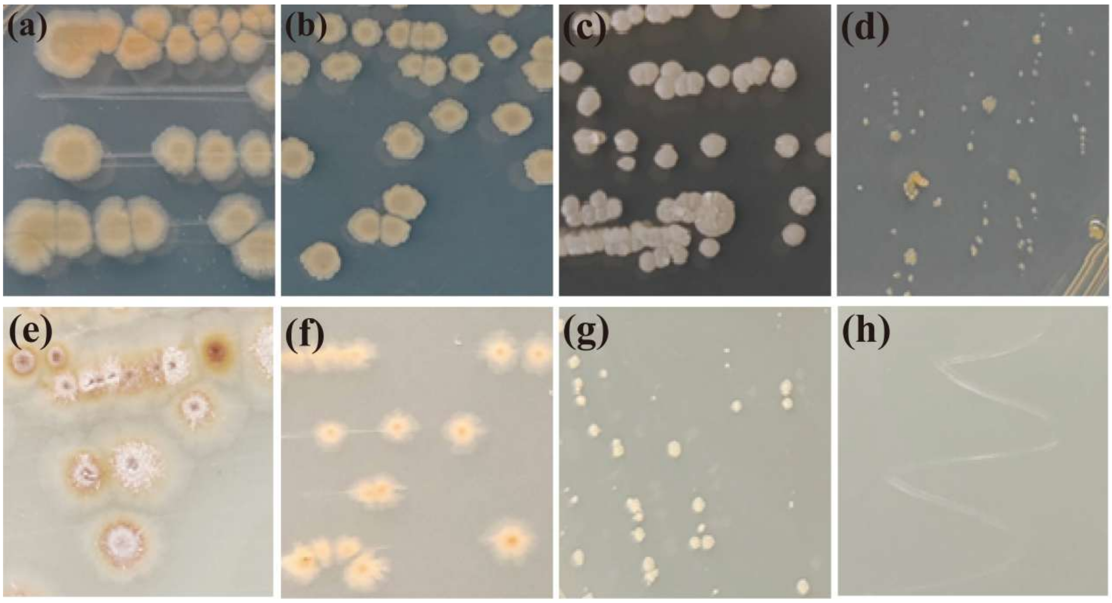

Figure S2. The colony morphology of strain HC307^T on eight mediums after being incubated at 28 °C for 7 days; (a) Gauze's medium No.1; (b) Czapek-Dox agar; (c) ISP 1; (d) ISP 2; (e) ISP 3; (f) ISP 4; (g) Bennett’s agar; (h) Potato dextrose agar.

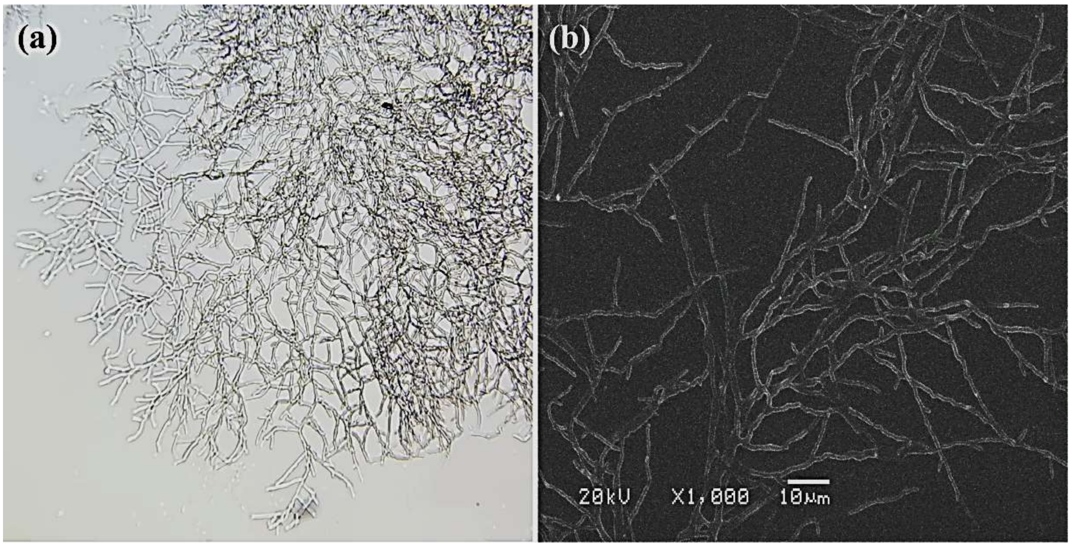

Figure S3. The morphology of hyphae of strain HC307^T; (a) hyphae of HC307^T observed under 400-fold optical microscopy; (b) hyphae of HC307^T observed under scanning electron microscopy.

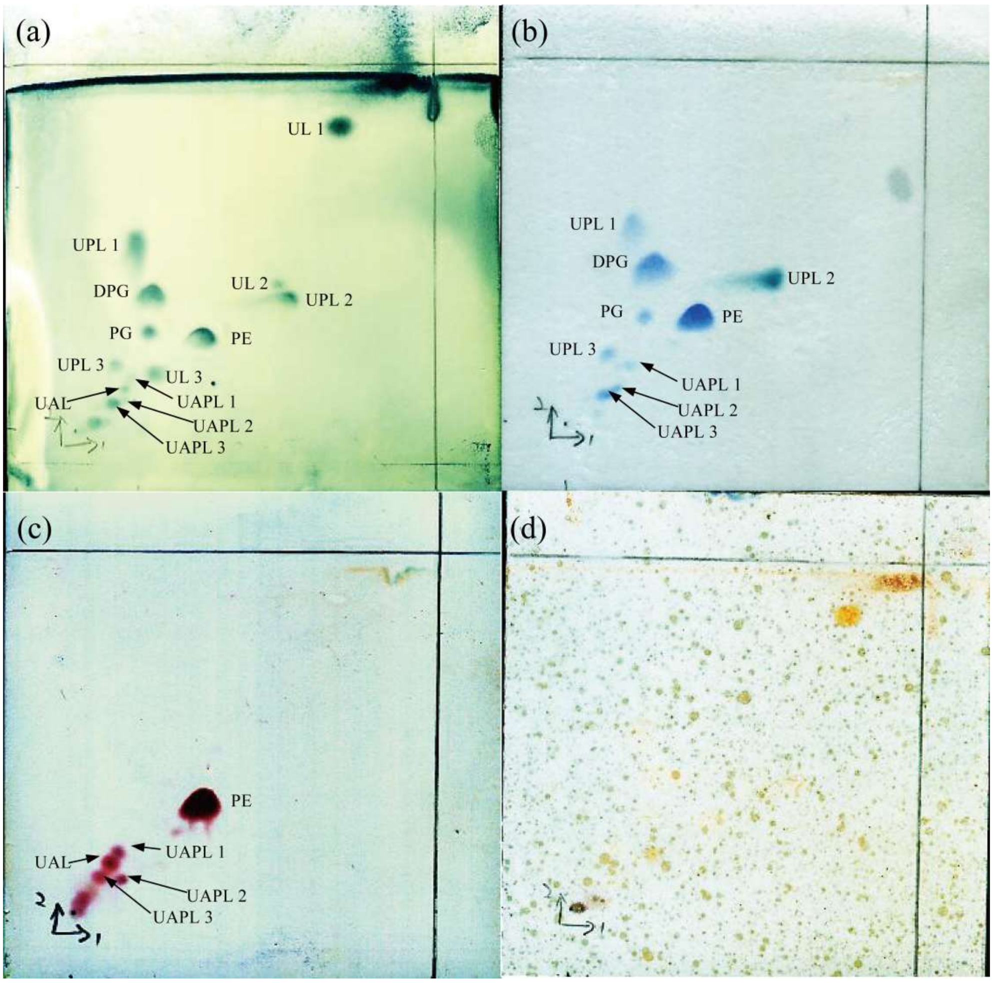

Figure S4. The polar lipid profile of strain HC307^T after separation by two-dimensional TLC; (a) Phosphomolybdic acid staining; (b) Molybdate staining; (c) Ninhydrin staining; (d) α-naphthol staining; DPG, diphosphatidyl glycerol; PE, phospatidylethanolamine; PG, phosphatidyl glycerol; UAPL1-3, unidentified aminophospholipids; UPL1-3, unidentified phospholipids; UAL, unidentified aminolipid; UL1-3, unidentified lipids.

2. Supplementary Tables

Table S1. The draft genomic features of strain HC307^T are described.

Characteristic	Number
Genome status	draft
Genome size (bp)	10,026,667
Number of contigs	2
Contig N50 (bp)	10,014,678
N90 length (bp)	10,014,678
G+C content (mol%)	70.03
Geneset number	9377
Genes assigned to:	
rRNA (5S, 16S, 23S)	18 (6,6,6)
tRNA	78
KEGG	2,895
GO	6,160
eggNOG	6843
Swiss-Prot	3983
Pfam	7065
CARD	2
CAZy	418
GenBank accession number	CP176503-CP176504

Table S2. Pairwise comparisons of the genome of strain HC307^{T} (PQ738958) versus type strain genomes with TYGS (https://tygs.dsmz.de/) accessed on 18 October 2024 and ANI (www.ezbiocloud.net/tools/ani) accessed on 17 October 2024.

**Type strain**	**GenBank**	**dDDH (d_4, %)**	**CI (d4, %)**	**ANI value (%)**	**G+C content difference (%)**
Streptomyces spinoverrucosus NBRC 14228^{T}	GCA_006539505.1	33.9	[31.4 - 36.4]	87.49	0.67
Streptomyces gossypiisoli TRM 44567^{T}	GCA_013433285.1	33.8	[31.3 - 36.3]	87.21	0.74
Streptomyces spinoverrucosus JCM 5077^{T}	GCA_014651195.1	33.8	[31.4 - 36.3]	87.54	0.66
Streptomyces cadmiisoli ZFG47^{T}	GCA_003261055.1	33.3	[30.9 - 35.8]	86.91	0.73
Streptomyces chromofuscus DSM 40273^{T}	GCA_015160875.1	32.6	[30.1 - 35.1]	86.64	1.58
Streptomyces curacoi DSM 40107^{T}	GCA_001513975.1	29.1	[26.7 - 31.6]	84.78	0.96
Streptomyces lomondensis JCM 4866^{T}	GCA_014651035.1	28.5	[26.1 - 31.0]	84.60	1.69
Streptomyces caelestis DSM 40084^{T}	GCA_014205255.1	28.4	[26.0 - 30.9]	84.34	0.95
Streptomyces caelestis JCM 4566^{T}	GCA_014650295.1	28.3	[25.9 - 30.8]	84.24	0.96
Streptomyces cylindrosporus 7R015^{T}	GCA_022698165.1	27.7	[25.3 - 30.2]	83.76	0.99
Streptomyces flaveolus JCM 4032^{T}	GCA_014648815.1	27.5	[25.1 - 30.0]	83.87	2.22
Streptomyces coelescens JCM 4739^{T}	GCA_014650795.1	26.5	[24.1 - 28.9]	82.88	2.22
Streptomyces humiferus JCM 3037^{T}	GCA_014647695.1	26.4	[24.1 - 28.9]	82.90	1.9
Streptomyces anthocyanicus JCM 5058^{T}	GCA_014651155.1	26.4	[24.1 - 28.9]	82.95	2.32
Streptomyces rubradiris JCM 4955^{T}	GCA_014656255.1	26.4	[24.1 - 28.9]	82.60	2.36
Streptomyces chiangmaiensis TA4-1^{T}	GCA_036281785.1	25.8	[23.5 - 28.3]	82.16	0.29
Streptomyces matensis JCM 4277^{T}	GCA_014649175.1	25.6	[23.3 - 28.1]	82.26	2.59
Streptomyces intermedius JCM 4483^{T}	GCA_039532785.1	22.6	[20.3 - 25.0]	78.25	3.04

Table S3. Culture characteristics of the strain HC307^{T} in 28 °C for 7 days.

Medium	Aerial mycelium	Vegetative mycelium	Soluble pigment	Growth situation
Gauze's medium No.1	Yellow	Brownish yellow	Pink	++
Czapek-Dox agar	Yellow	Brownish yellow	/	+
ISP 1	Rice powder pink	Yellow	/	+
ISP 2	Beige	Beige	/	(+)
ISP 3	Pink	Pink	/	++
ISP 4	Orange circles	Pale yellow	/	+
Bennett's agar	Pale yellow	Pale yellow	/	(+)
Potato dextrose agar	/	/	/	-

++, good; +, positive; (+), weakly positive; -, negative; /, no result.

Table S4. The analysis of biosynthetic pathways in strain HC307^T by antiSMASH 7.1.0.

Cluster	Type	Location (nt)	Predicted product	Similarity (%)
1.1	T3PKS	50,541 - 91,641	Alkylresorcinol	100
1.2	NAPAA	255,570 - 291,220	Stenothricin	13
1.3	Terpene	371,745 - 393,631	Paulomycin	7
1.4	RiPP-like	592,247 - 602,462	Informatipeptin	42
1.5	Terpene	621,504 - 642,535	Cyphomycin	2
1.6	Terpene	648,699 - 669,385	Unknown	---
1.7	T1PKS	1,146,050 - 1,189,124	4-hexadecanoyl-3-hydroxy-2-(hydroxymethyl)-2H-furan-5-one	90
1.8	NRPS, terpene	1,262,643 - 1,334,973	Hopene	92
1.9	Hydrogen-cyanide	1,406,105 - 1,418,966	Aborycin	28
1.10	Indole	1,841,193 - 1,862,344	5-isoprenylindole-3-carboxylate β-D-glycosy ester	28
1.11	NI-siderophoree	1,968,444 - 1,999,555	Paulomycin	11
1.12	NRP-metallophore, NRPS	2,052,968 - 2,111,357	Coelichelin	100
1.13	Terpene, butyrolactone	2,300,346 - 2,323,402	γ-butyrolactone	100
1.14	RiPP-like	2,376,972 - 2,388,270	Unknown	---
1.15	T2PKS	2,551,836 - 2,624,335	Allocyclinone	59
1.16	NI-siderophore	2,702,729 - 2,732,744	Kinamycin	16
1.17	Triceptide	3,359,612 - 3,380,972	Unknown	---
1.18	Terpene	3,433,462 - 3,454,475	Albaflavenone	100
1.19	Triceptide, lanthipeptide-class-i	4,522,745 - 4,550,678	Unknown	---
1.20	T3PKS	5,179,793 - 5,220,857	7-deoxypactamycin	18
1.21	NAPAA	6,267,119 - 6,303,118	Stenothricin	13
1.22	NI-siderophore	6,327,328 - 6,357,097	Desferrioxamin B/desferrioxamine E	100
1.23	Melanin	6,439,734 - 6,450,243	Istamycin	4
1.24	T3PKS	6,786,577 - 6,827,662	S56-p1	11
1.25	Ectoine	7,540,305 - 7,550,709	Ectoine	100
1.26	NAPAA	7,806,453 - 7,840,319	$\mathcal{E}$-poly-L-lysine	100
1.27	NRPS-like, NRPS	8,336,147 - 8,398,185	Himastain	16
1.28	T1PKS	8,524,004 - 8,579,485	Neocarzin A/neocarzin B	100
1.29	Indole	8,655,220 - 8,675,936	Griseusin	15
1.30	Phosphonate	8,866,803 - 8,881,544	Deoxyhangtaimycin	2
1.31	T2PKS	9,479,799 - 9,552,314	Spore pigment	83
1.32	RiPP-like	9,744,546 - 9,755,925	Streptamidine	75

---, no result of prediction.
